# Supplementary material for: Hepatocyte-Specific Phgdh-Deficient Mice Culminate in Mild Obesity, Insulin Resistance, and Enhanced Vulnerability to Protein Starvation
Source: Nutrients. 2021 Sep 29;13(10):3468. doi: 10.3390/nu13103468 (PMC8537398; doi:10.3390/nu13103468)
Supplement: Supplementary file 1 [file nutrients-13-03468-s001.zip › nutrients-1391596-supplementary.pdf]

Hepatocyte-specific *Phgdh* deficient mice culminate in mild obesity, insulin resistance, and enhanced vulnerability to protein starvation

Momoko Hamano, Kayoko Esaki, Kazuki Moriyasu, Tokio Yasuda, Sinya Mori, Kosuke Tashiro, Yoshio Hirabayashi and Shigeki Furuya

Supplementary Table S1

Amino acid concentration in serum

amino acid	concentration (nmol/g weight tissue) Floxed group	LKO group	ratio (%: LKO/Floxed)	*p*-value
L-Asp	5.85 ± 1.63	12.86 ± 3.53	219.8	0.10
L-Glu	108.96 ± 14.32	110.55 ± 14.49	101.5	N.S.
L-Ser	110.49 ± 17.05	130.07 ± 18.16	117.7	N.S.
L-Gln	540.12 ± 33.53	537.25 ± 28.85	99.5	N.S.
L-His	46.51 ± 5.63	52.54 ± 5.73	113.0	N.S.
L-Thr	178.11 ± 29.01	154.8 ± 11.73	86.9	N.S.
Gly	427.79 ± 33.71	467.88 ± 30.95	109.4	N.S.
L-Arg	199.08 ± 34.16	197.74 ± 17.94	99.3	N.S.
Tau	1187.01 ± 97.94	1136.12 ± 59.17	95.7	N.S.
GABA	N.D.	N.D.		
L-Ala	429.67 ± 56.47	411 ± 34.81	95.7	N.S.
L-Tyr	61.92 ± 14.38	64.57 ± 12.54	104.3	N.S.
L-Val	117.11 ± 18.51	100.33 ± 19.92	85.7	N.S.
L-Met	27.98 ± 7.52	30.06 ± 7.05	107.4	N.S.
L-Phe	94.58 ± 13.14	89.65 ± 7.4	94.8	N.S.
L-Ile	54.64 ± 10.92	48.72 ± 4.54	89.2	N.S.
L-Leu	418.25 ± 66.56	393.54 ± 18.32	94.1	N.S.

Supplementary Figure S1

Floxed LKO

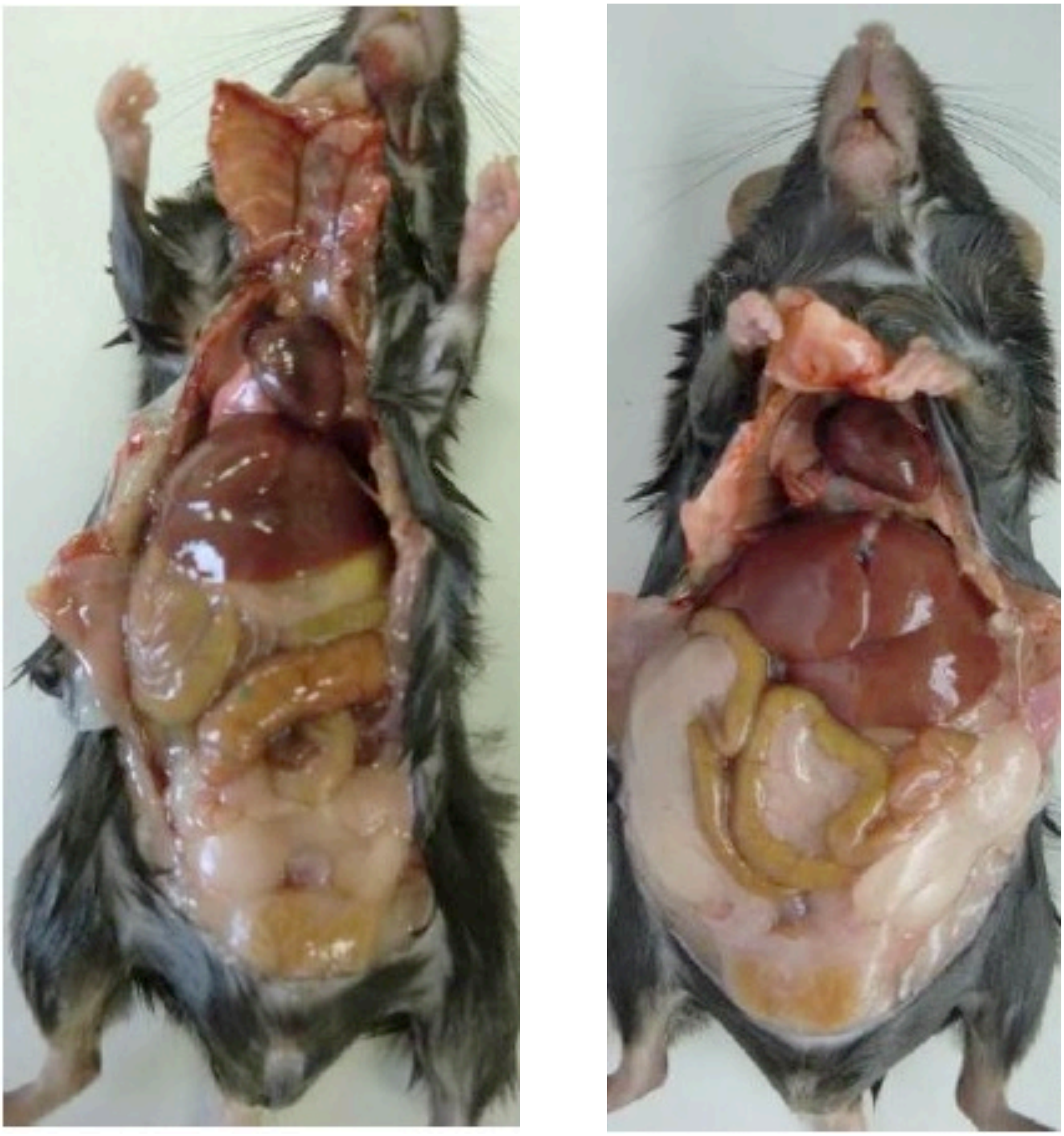

Supplementary Figure S1

Floxed LKO

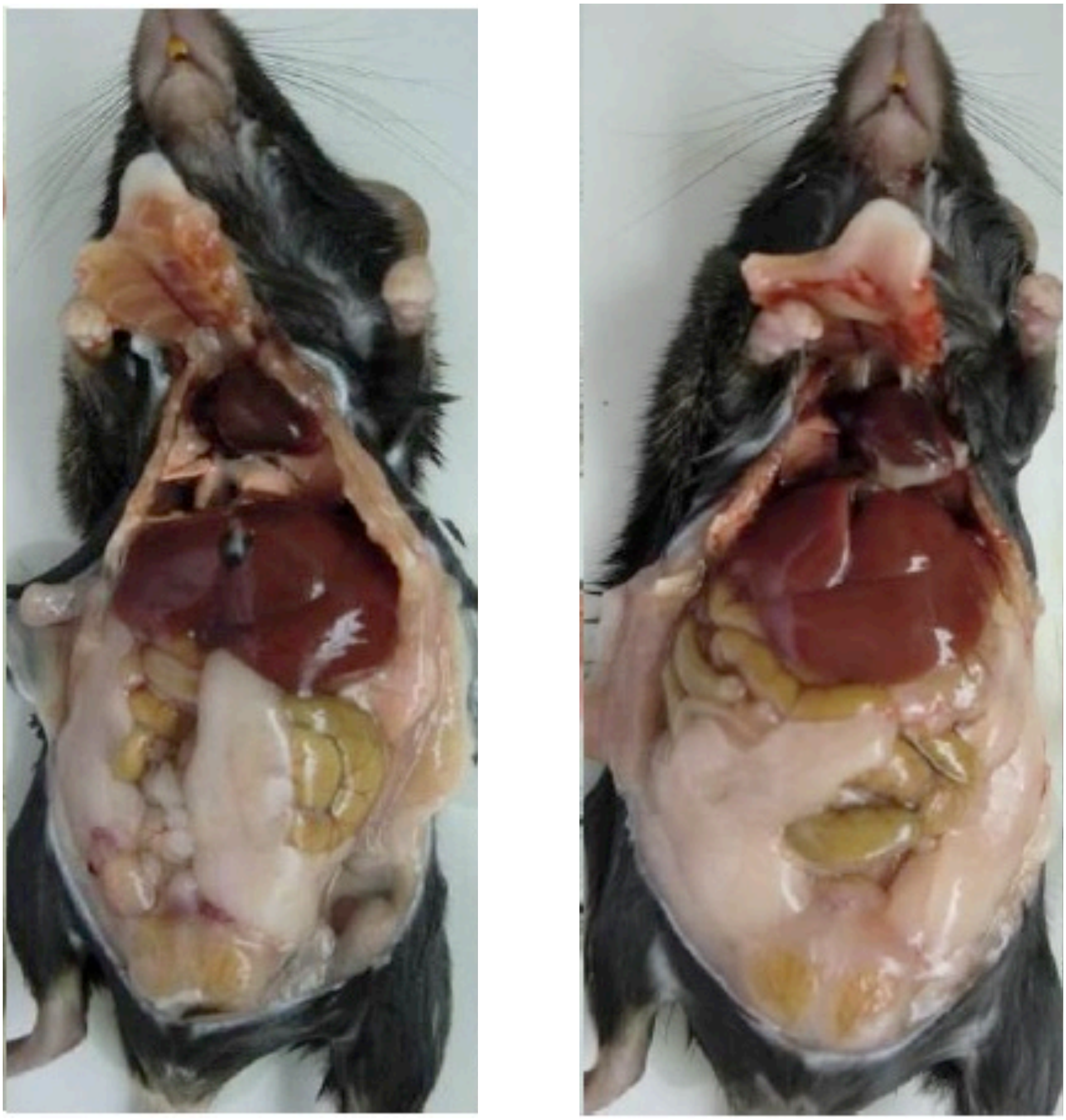

Supplementary Figure S1

Floxed LKO

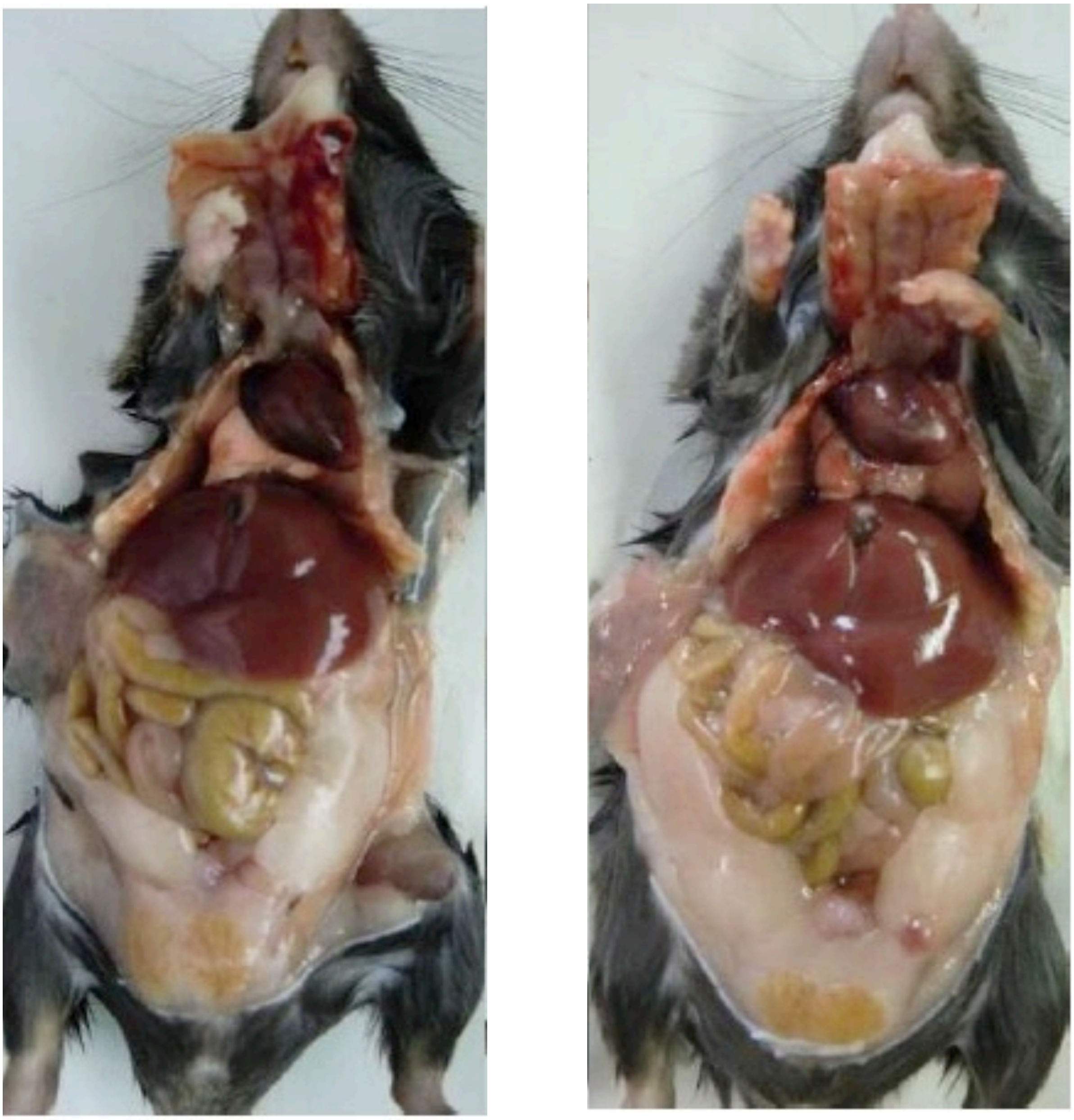

Supplementary Figure S2

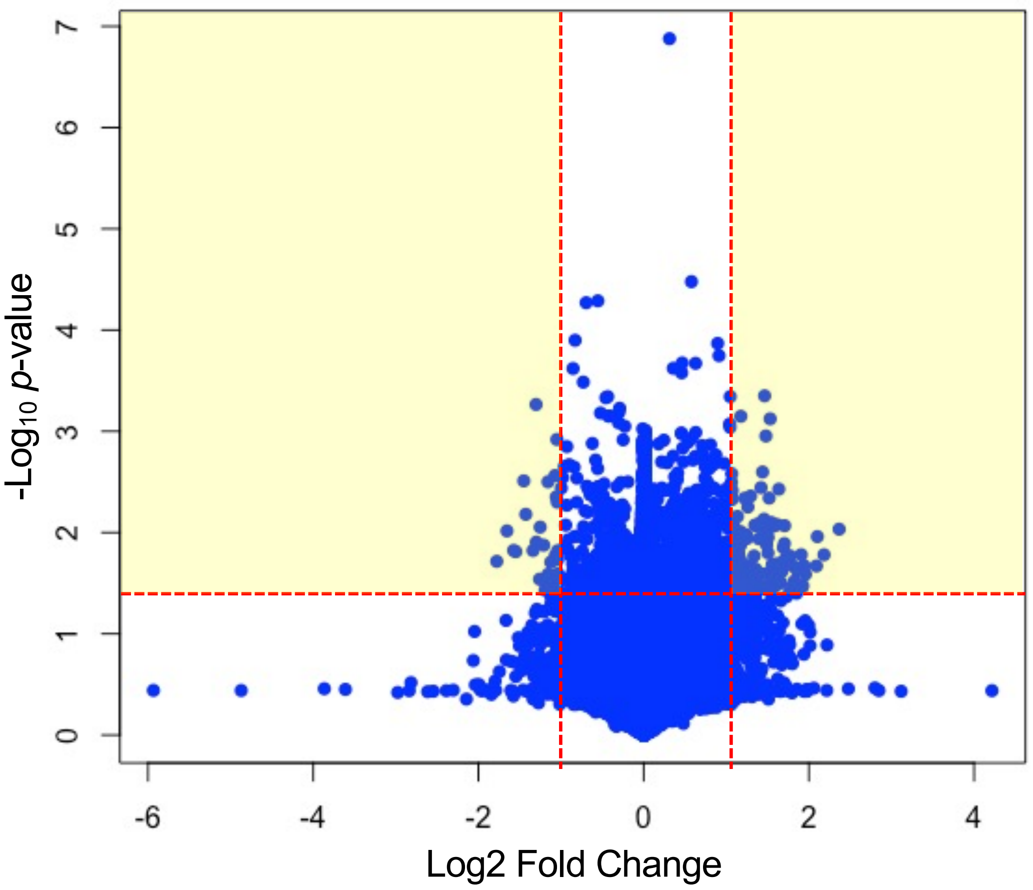

DEGs were selected with thresholds of
$|\log_2\text{Fold change}| \geq 1$and $P < 0.05$.
Statistical analysis was performed using the Student's t-test.

Supplementary Figure S3

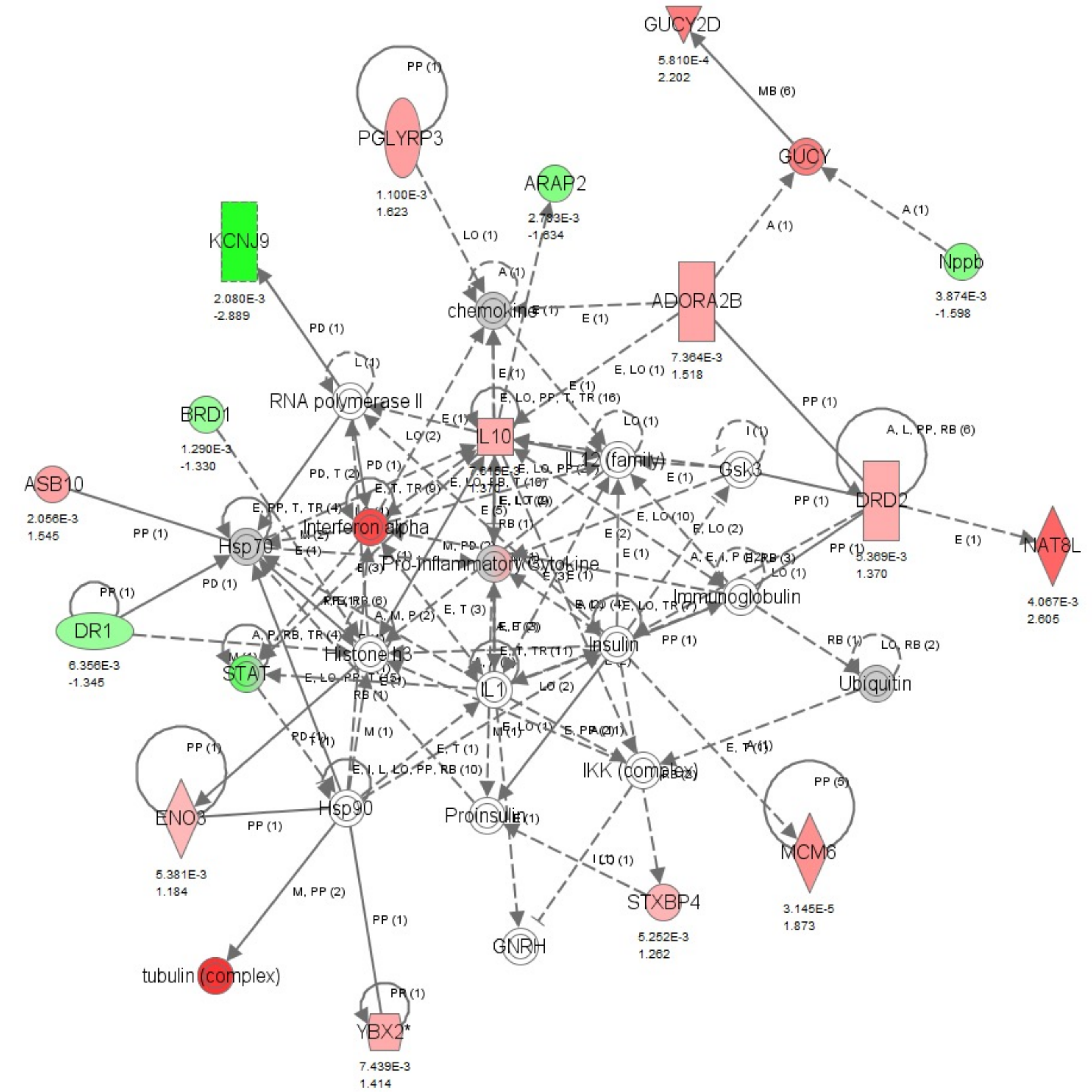

The network is displayed graphically as nodes (genes/proteins) and edges (biological interactions between the nodes). The node color intensity indicates the degree of down- (green) or upregulation (red). Nodes are displayed using various shapes representing the functional class of the gene product. Edges are displayed with various labels that present the biological nature of interactions between the nodes as follows: A, activation; B, binding; E, expression; I, inhibition; LO, localization; P, phosphorylation/dephosphorylation; PD, protein–DNA binding; PR, protein–mRNA binding; PP, protein–protein binding; T, transcription. Straight lines indicate direct interactions, and dashed lines indicate indirect interactions. Edges without a label represent binding only.
